# Carbimazole Induced Pleural Effusion: A Case Report

**DOI:** 10.1155/2012/941241

**Published:** 2012-03-29

**Authors:** Gautam Das, Stephen E. R. S. Stanaway, Liz Brohan

**Affiliations:** ^1^Department of Diabetes and Endocrinology, Glan Clwyd Hospital, Rhuddlan Road, Bodelwyddan, Rhyl LL18 5UJ, UK; ^2^Department of Diabetes and Endocrinology, Wrexham Maelor Hospital, Croesnewyyd Road, Wrexham LL13 7TD, UK; ^3^Department of Respiratory Medicine, Wrexham Maelor Hospital, Croesnewyyd Road, Wrexham LL13 7TD, UK

## Abstract

*Objective*. To describe a patient with unilateral exudative pleural effusion that developed after commencement of carbimazole. *Methods*. We describe the presentation and clinical journey of an elderly woman who presented to the chest physicians initially with pleural effusion but was followed up by the endocrinology team. *Result*. The patient was a 77-year-old Caucasian woman who presented with symptoms of breathlessness and a confirmed unilateral pleural effusion while being on treatment for thyrotoxicosis. Her symptoms needed recurrent hospital admission for investigations and drainage, but no potential cause was identified after extensive investigations. A drug-induced exudative effusion consequent to carbimazole intake was diagnosed as discontinuation of the drug lead to complete resolution of the effusion with no recurrence. *Conclusion*. Physicians and Endocrinologist must bear in mind that this potentially rare complication of carbimazole while treating patients of thyrotoxicosis as appearance of similar features in their patients while being on carbimazole should lead to the discontinuation of the drug, and alternative treatment strategy should be considered.

## 1. Introduction

A myriad of complications are associated with antithyroid medications, but pleuropulmonary complications are not commonly encountered. ANCA-positive vasculitis in association with antithyroid drugs has been described since 1992 [1], and 27% of patients may present with respiratory tract involvement [2]. Most of the cases are predominantly linked to propylthiouracil, but sporadic cases of lung involvement due to methimazole have also been reported [3]. Interestingly there is no much published data regarding patients who are ANCA negative and develop systemic vasculitis or serositis leading to third space collections in relation to carbimazole or methimazole. We hereby present the case of an elderly woman who developed an exudative pleural effusion after a short duration of carbimazole treatment and had a negative immunology. We suspect that this is due to a local inflammation or vasculitic reaction in the pleural space although a conclusive explanation is elusive. 

## 2. Presentation of the Case

A 77-year-old female was admitted with symptoms of weight loss, increased shortness of breath, and palpitation. She had a new onset atrial fibrillation on her ECG with evidence of minimal bilateral pleural effusion on a chest X-ray, and her routine bloods were fairly unremarkable except a thyrotoxic picture with an FT3 of 13 pmol/L and a suppressed TSH of 0.07 *μ*U/mL. Her antithyroid antibodies (thyroperoxidase and TSH receptor) were negative, and a thyroid ultrasound showed a multinodular goiter. She also had a CT pulmonary angiogram looking into her breathlessness which confirmed a left-sided pulmonary embolism, and a consequent echocardiogram showed an ejection fraction of 30%. She was managed with diuretics and was commenced on carbimazole, propranolol, and warfarin. She recovered very well but couldn't tolerate propranolol and was commenced on bisoprolol as an alternative. Her other medications at that point were frusemide and lansoprazole only. She was discharged home with a provisional diagnosis of congestive cardiac failure secondary to pulmonary embolism and atrial fibrillation and hyperthyroidism.

She was seen in the clinic 3 months after her discharge with complaints of breathlessness and left posterior pleuritic type chest pain. Examination confirmed dullness on the left side, and a chest X-ray revealed a massive unilateral left sided pleural effusion. Her thyroid function had reasonably settled by then with a FT3 of 3.8 pmol/L, and the TSH was 0.03 *μ*U/mL. This presentation was different to her last when she had minimal bilateral pleural effusion consequent to heart failure hence it was decided to admit her electively for further investigations. During hospital stay her thyroid function had settled further (FT4 5.8 pmol/L, FT3 3.4 pmol/L and TSH recovered to 2.1 *μ*U/mL) being on Carbimazole 20 mg alone. A CT scan showed unilateral left-sided effusion with no mass lesion or adenopathy. Pleural fluid aspiration confirmed and exudate with mesothelial cells only and the gram stain, AAFB stain, and culture was negative. Her fluid was therapeutically drained following which she had a thoracoscopy which only showed benign looking pleura, and biopsy confirmed inflammatory changes. In view of her low FT4, she was commenced on thyroxine 50 *μ*g once daily as a block and replace regime with carbimazole and was sent home with the view of arranging further tests as an outpatient.

**Figure 1:**
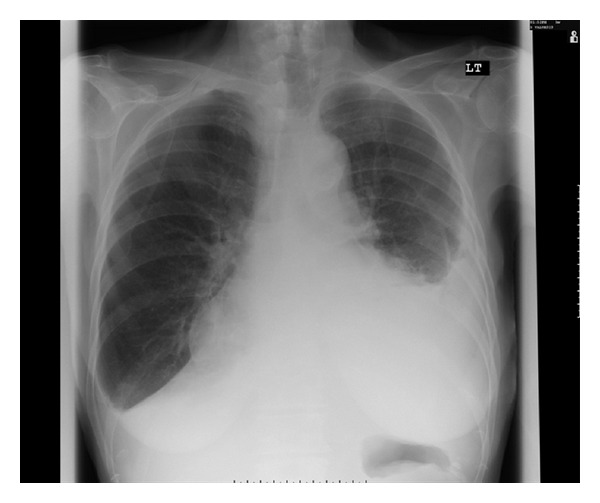
Pleural effusion while being on carbimazole.

She was admitted again as an emergency in the next few weeks with a recollection of her left pleural effusion (Figure 1). She was then actively investigated in view of searching for an occult malignancy which would explain her weight loss and effusion as she had a history of smoking in the past. A CT chest abdomen pelvis only confirmed the effusion with no other positive findings. A diagnostic aspiration confirmed an exudate again with no positive cytology. Her myeloma screen, vasculitic screen, immunoglobulin's, and serum tumor markers were all within normal limits. An upper gastrointestinal endoscopy for investigating her weight loss showed three gastric ulcers, but her gut hormone profile (VIP, gastrin, and chromagranin) was within normal limits. A bronchoscopy carried out at this stage also did not reveal any answers into the cause of her pleural effusion. She did not have any infective symptoms all this while the markers of inflammation (white cells and CRP) were all within normal limits. Her drug history was revisited at that point, and it was felt whether carbimazole could be a potential cause of her effusion although this was not mentioned in the British National formulary which we follow as a standard in the United Kingdom. Her carbimazole was replaced with propylthiouracil, and she was sent home with the plan of getting her admitted again for a pleurodesis or tunnel drainage if there was any further recurrence.

**Figure 2:**
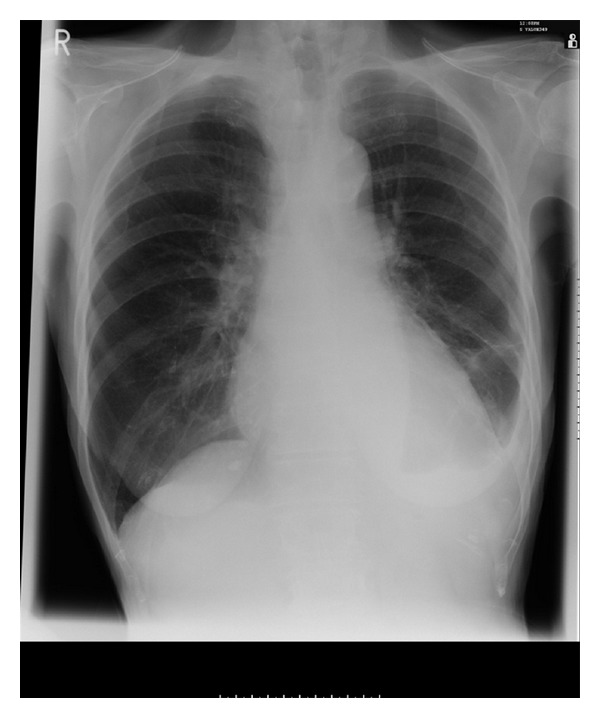
Resolution of pleural effusion after discontinuation of carbimazole.

Fortunately, we did not hear from the patient again in the next few months, and she was routinely seen in the clinic after three months. We were pleased to note that she was symptomatically much better and looked clinically and biochemically euthyroid. She managed to gain some weight, and a repeat chest X-ray showed a complete resolution of her effusion with no evidence of recurrence (Figure 2).

## 3. Discussion

Several adverse effects are related to antithyroid medications although pleural and pulmonary complications are usually rare in relation to these agents. Pulmonary involvement associated with vasculitis and a positive ANCA secondary to antithyroid medication are known [2] but the majority of them has been with propylthiouracil, and carbimazole-induced vasculitis is exceedingly rare as till date only nine cases have been reported [4–6]. Patients usually present with pulmonary capillaritis, intra-alveolar hemorrhage, and respiratory failure [7], but reports suggest that eosinophilic pleural effusion may be encountered due to propylthiouracil alone [8, 9]. Several drugs have been known to cause exudative pleural effusion [10, 11], but there is no such reports in the literature implicating carbimazole as the offender as per our knowledge. A possible mechanism could be due to local pleuritis or leukocytoclastic vasculitis, but it remains to be proved. There are no data to suggest whether carbimazole can alter the configuration of myeloperoxidase [2] leading to significant ANCA negativity in most cases, hence it could be assumed that patients may have vasculitic features with serosal involvement without a positive immunology when treated with carbimazole. It is a matter of debate whether the unilateral exudative effusion in our patient could be explained by a similar mechanism, but our argument is based on the fact that her symptoms of unilateral effusion started only after carbimazole was commenced, and all potential causes for an exudative effusion were excluded by thorough investigations, and there was a complete resolution of the effusion after cessation of carbimazole therapy. It has been suggested by other authors in the past that if the cause of exudative pleural effusion is not clinically obvious, then drug therapy withdrawal should be considered [11] and that is precisely what was done for this patient.

## 4. Conclusion

In conclusion, this case illustrates the importance of being aware of the relatively rare and not so well-known adverse effect of carbimazole in relation to pulmonary disease. Stopping the medicine with resolution of symptoms proves the diagnosis, but the mechanism remains unclear in patients who are ANCA negative. Development of such complications limits the use of alternative such as propylthiouracil; hence, patients should be considered for definitive treatment like radioiodine or surgery in such circumstances.
